# DNA methylation and transcriptomic features are preserved throughout disease recurrence and chemoresistance in high grade serous ovarian cancers

**DOI:** 10.1186/s13046-022-02440-z

**Published:** 2022-07-27

**Authors:** Nicole Gull, Michelle R. Jones, Pei-Chen Peng, Simon G. Coetzee, Tiago C. Silva, Jasmine T. Plummer, Alberto Luiz P. Reyes, Brian D. Davis, Stephanie S. Chen, Kate Lawrenson, Jenny Lester, Christine Walsh, Bobbie J. Rimel, Andrew J. Li, Ilana Cass, Yonatan Berg, John-Paul B. Govindavari, Joanna K. L. Rutgers, Benjamin P. Berman, Beth Y. Karlan, Simon A. Gayther

**Affiliations:** 1 Department of Biomedical Sciences, Center for Bioinformatics and Functional Genomics, Cedars-Sinai Medical Center, Los Angeles, CA 90048 USA; 2 Division of Biostatistics, Department of Public Health Sciences, University of Miami, Miller School of Medicine, Miami, FL 33101 USA; 3 Applied Genomics, Computation and Translational Core, Cedars Sinai Medical Center, Los Angeles, CA 90048 USA; 4 Women’s Cancer Program, Samuel Oschin Comprehensive Cancer Institute, Cedars-Sinai Medical Center, Los Angeles, CA 90048 USA; 5 Division of Gynecologic Oncology, Department of Obstetrics and Gynecology, Cedars-Sinai Medical Center, Los Angeles, CA 90048 USA; 6 Department of Obstetrics and Gynecology, David Geffen School of Medicine, Jonsson Comprehensive Cancer Center, UCLA, Los Angeles, CA 90048 USA; 7 Division of Gynecologic Oncology, Department of Obstetrics and Gynecology, Dartmouth-Hitchcock Medical Center, Hanover, NH 03756 USA; 8 Department of Developmental Biology and Cancer Research, Institute for Medical Research Israel-Canada, Hebrew University-Hadassah Medical School, 91120 Jerusalem, Israel; 9 Department of Pathology, Cedars-Sinai Medical Center, Los Angeles, CA 90048 USA

**Keywords:** High grade serous ovarian cancer, Methylation, Chemoresistance, Epigenetics, Whole genome bisulfite sequencing, Computational methods, Translational research

## Abstract

**Background:**

Little is known about the role of global DNA methylation in recurrence and chemoresistance of high grade serous ovarian cancer (HGSOC).

**Methods:**

We performed whole genome bisulfite sequencing and transcriptome sequencing in 62 primary and recurrent tumors from 28 patients with stage III/IV HGSOC, of which 11 patients carried germline, pathogenic *BRCA1* and/or *BRCA2* mutations.

**Results:**

Landscapes of genome-wide methylation (on average 24.2 million CpGs per tumor) and transcriptomes in primary and recurrent tumors showed extensive heterogeneity between patients but were highly preserved in tumors from the same patient. We identified significant differences in the burden of differentially methylated regions (DMRs) in tumors from *BRCA1/2* compared to non-*BRCA1/2* carriers (mean 659 DMRs and 388 DMRs in paired comparisons respectively). We identified overexpression of immune pathways in *BRCA1/2* carriers compared to non-carriers, implicating an increased immune response in improved survival (*P* = 0.006) in these *BRCA1/2* carriers.

**Conclusion:**

These findings indicate methylome and gene expression programs established in the primary tumor are conserved throughout disease progression, even after extensive chemotherapy treatment, and that changes in methylation and gene expression are unlikely to serve as drivers for chemoresistance in HGSOC.

**Supplementary Information:**

The online version contains supplementary material available at 10.1186/s13046-022-02440-z.

## Background

Studies of the methylome and transcriptome in cancer have shown that conserved somatic alterations can disrupt the molecular pathways that precede the onset of neoplasia and contribute to the development and progression of cancer and chemoresistance. The development of methods for large scale genomic analyses of cancers has established that DNA methylation and its role in regulating gene expression is a key component in the development of most solid tumors. Germline or somatic hypermethylation is often an alternative mechanism to pathogenic loss of function mutations caused by coding and splice site mutations, deletions or rearrangements that lead to allele specific gene deregulation. Hypermethylation silences genes that are critical components of genome integrity (e.g., DNA repair genes, cell cycle regulation genes and tumor suppressor genes) and may be an early event in the progression to cancer. Indeed, loss of expression of genes involved in cancer development occurs about 10 times more frequently by DNA hypermethylation of promoter CpG islands than through mutation of DNA [1–3].

Initial studies focused on the role of methylation within CpG islands of gene promoters as a mechanism to silence gene expression [4–6], identifying key driver events in known cancer associated pathways. For example, somatic changes in methylation at the *BRCA1* promoter, result in reduced expression of *BRCA1*, homologous DNA repair deficiency [7, 8] and shorter survival [9] in primary ovarian cancers. Alterations in methylation in the promoters of oncogenes (*PIK3CA*), transcription factors (*WT1*) and driver genes (*TP53*) have also been implicated in ovarian cancer development [10]. Together, these data support a role of methylation in the regulation of genes that are critical in tumor initiation and development. Methylation is a genome-wide phenomenon that also targets the promoters of non-coding RNAs [11, 12] and more distal regulatory elements such as enhancers [13, 14]. Thus, DNA methylation can contribute to aberrant gene expression by altering the activity of transcription factor binding sites within enhancers and critical networks of gene expression variation involved in disease pathogenesis. More recent studies have shown that a global loss of methylation occurs in cancers, likely as part of the mitotic clock, across broad regions of the genome, known as partially methylated domains (PMDs) [13, 15–18]. These regions generally harbor genes expressed at low levels and account for the bulk of methylation changes that occur in cancer [13, 15, 16, 18, 19].

Array based methods have become commonplace tools to evaluate the contribution of methylation to tumor pathogenesis. However, even the latest arrays measure the methylation status of only a small fraction of the nearly 30 million known CpGs throughout the genome (e.g., the latest iteration, the Illumina MethylationEPIC BeadChip array covers 850,000 CpG sites). Recent advances in whole genome bisulfite sequencing (WGBS) for methylation profiling provide single-base resolution, expanding our ability to identify functionally relevant DNA methylation regions on the basis of transcriptional regulation. WGBS analyses have not yet been performed in substantially large numbers of tumors, but the method is already providing novel insights into the role of methylation in cancer.

The contribution of DNA methylation to tumor recurrence and chemoresistance in ovarian cancer is poorly understood. Despite early indications that demethylating agents may be effective treatments for high grade serous ovarian cancer (HGSOC) [20] recent clinical trials of hypomethylating agents, including guadecitabine, have yielded little improvement in survival or resensitization to platinum-based therapies [21, 22]. HGSOC is the most common and lethal histotype of ovarian cancer. About 70% of affected women are diagnosed with advanced stage disease (stages III/IV) and of these women < 30% will survive more than five years. Patients are treated with maximal debulking surgery followed by combination chemotherapy with platinum. Typically, patients initially respond well to treatment, but usually relapse with recurrent and eventually chemoresistant disease [23]. Between 15 and 25% of tumors are classified with primary resistance [24]. This tends to occur in patients with homologous recombination proficient tumors and/or amplification of the *CCNE1* locus at 19q12 [7, 8]. Nearly a third of all HGSOC cases have germline or somatic alterations in the *BRCA1* or *BRCA2* genes [7, 8], which result in DNA double strand break repair deficiency and an accumulation of DNA damage as tumors develop. The goals of this study were: (1) to establish the underlying role of DNA methylation in the recurrence and chemoresistance of HGSOC; and (2) to identify the role of DNA methylation in the development of HGSOC in women with and without germline defects in *BRCA1* and *BRCA2*.

## Methods

### Experimental Design

#### Cohort description

Fresh-frozen matched primary and recurrent high-grade serous ovarian cancer specimens and DNA from twenty-seven consented women diagnosed with high grade serous adenocarcinoma were included, as well as one additional primary tumor specimen with no matched recurrent tumor (28 patients total). Specimens were all identified in the Cedars-Sinai Medical Center Women’s Cancer Program Biorepository (IRB #0901). 11 of these women had deleterious *BRCA1* and/or *BRCA2* germline mutations and 17 did not harbor any known high or moderate risk mutations for HGSOC (*BRCA1, BRCA2, RAD51C, RAD51D, BRIP1* and *FANCM*) identified in clinical genetic testing. All patients were diagnosed with stage III or stage IV disease and underwent primary optimal surgical cytoreduction (to less than 1 cm residual disease) prior to administration of combination chemotherapy with platinum and taxane between the years of 1990 and 2014. For each patient, detailed clinical data were available including clinical genetic testing results, dates of original diagnosis and each subsequent recurrence, treatments administered throughout their disease course, operative and pathology reports, and other clinicopathologic variables including other cancer diagnoses and comorbidities. These details can be found in Additional File 1, Supplementary Table 1.

#### Specimen acquisition and preparation

Fresh frozen tumors were embedded in optimal cutting temperature (OCT) compound, bisected and mounted and two slides were made for hematoxylin and eosin (H&E) staining. All slides were reviewed by a single pathologist to identify regions enriched for epithelial carcinoma (avoiding tumor stroma), which are then collected in a single punch of approximately 50 mg collected on dry ice. Each punch was divided into three pieces, two of which were used for genomic DNA (gDNA) extraction using the Machery-Nagel Nucleospin DNA Kit, and the third for RNA extraction using the Machery-Nagel Nucleospin RNA Kit. DNA samples were assayed for quality using the QuBit (Thermo Fisher Sci, CA) to measure the content of double stranded DNA and by running 1ul gDNA on a 1.5% agarose gel at 100 V for 1 h to confirm no fragmentation of material has occurred during the extraction. RNA was extracted using an isopropyl-alcohol:chloroform approach following the standard operating protocol published by the Prostate Cancer Biorepository Network [25]. RNA was quantified on the Qubit in RNA mode to measure the amount of high quality dsRNA within the sample, and then on the Agilent Bioanalyzer, where an RNA Integrity Number (RIN) score is generated, reflecting the quality (by concentration and fragment size) of the samples. Germline DNA was extracted from whole blood drawn at the time of debulking surgery after diagnosis with HGSOC. DNA was extracted with the Qiagen DNEasy Blood & Tissue Kit (Qiagen, Germantown, MD, USA) and quantitated with the Quant-IT dsDNA Broad Range kit on a QuBit (Thermo Fisher, Waltham, MA, USA).

## Method details

### Whole genome bisulfite sequencing (WGBS)

Our workflow for WGBS required a minimum 300 ng of high quality gDNA, which was sheared to approximately 175–200 bp using a Covaris sonicator, and bisulfite converted using the EZ DNA Methylation-Lightning Kit (Zymo). Libraries were constructed using the Accel-NGS Methyl-Seq DNA Library Kit (Swift Biosciences, MI), and amplified using no more than 6 cycles of PCR. Libraries were sequenced to at least 30 × coverage (on average each base is sequenced thirty times) on the Illumina HiSeq4000 in 150 bp paired end mode. This approach generated approximately 400 million read pairs per library, with a bisulfite conversion rate greater than 99%.

### RNA-Seq library preparation and sequencing

RNA was extracted using the protocol published online by the Prostate Cancer Biorepository network (SOP#006), where frozen tissue was stored at -80C until extraction. 1 mL of trizol was added to the tissue in a 1.5 mL eppendorf tube and incubated for 5 min at 15-30C to dissociate nucleoprotein complexes. Next 200ul of chloroform was added and tubes capped and mixed vigorously for 15 secs then incubated at room temperature for 3 min. Samples were then centrifuged for 15 min at 4C at 12,000 g. The top, aqueous phase was removed to a fresh, sterile 1.5 ml tube and mixed with 500ul of isopropyl alcohol to precipitate the RNA. Samples were then incubated at room temperature for 10 min and then centrifuged for 10 min at 4C at 12,000 g. A pellet was visible in high yield samples, and supernatant was removed (and discarded), leaving the pellet untouched. The pellet was washed with 1 mL 75% ethanol and mixed by vortexing and then centrifuging at 7,500 g for 5 min at 4C. The supernatant was removed (leaving the pellet untouched) and the pellet allowed to air dry before being dissolved in 200ul RNase-free water and incubated at 55C for 10 min. Sample concentration was measured using the Qubit RNA Broad Range kit and sample quality was measured using the Agilent BioAnalyzer 2100. Sequencing libraries were prepared by adding 1ug of RNA to the TruSeq Stranded Total RNA Kit with Ribo- and Mito- depletion following the TruSeq standard protocol with 15 cycles of PCR. Libraries were quantified using Qubit RNA Broad Range kit and pooled before being run on one lane of a HiSeq2000 to collect ~ 1 M reads per library for quality control. PCR duplication rate was estimated in this low coverage sequencing run in 150 bp paired end mode, and library complexity was estimated using PreSeq. Based on the complexity measured in this low coverage sequencing experiment we estimated the maximal coverage that would continue to provide informative measurement of transcripts in the library was ~ 350 M reads. Each library was then pooled and this pool was sequenced in 2 × 150 bp mode on an Illumina Novaseq 6000, and we generated ~ 335 million reads from each library. Our data analysis workflow for RNA-Seq has been developed specifically to improve gene feature identification and measurement in archived frozen tissue samples, which can perform poorly using standardized workflows.

### Statistical analysis

#### WGBS data processing

WGBS reads were aligned to the human reference genome (build GRCh38) using BISCUIT [26]. Duplicate reads were marked using Picard Tools [27]. Methylation rates were called using BISCUIT. CpGs with fewer than 5 reads of coverage were excluded from further analysis. Adapter sequences were trimmed using TrimGalore [28], using default parameters for Illumina sequencing platforms. Quality control was performed using PicardTools as well as MultiQC [29]. Bisulfite non-conversion was checked using the Biscuit QC module in MultiQC. Principal Component (PC) analysis was performed on CpGs with coverage ≥ 10 and the top 10,000 most variable CpGs were included in the identification of the top 10 PCs using the prcomp function from the stats package in R [30].

#### Calling partially methylated domains (PMDs)

To call Partially Methylated Domains (PMDs), we first divided the genome of each sample into non-overlapping 100 kb bins, and took the average of all solo-WCGW CpGs within each bin, using the solo-WCGW definition from Zhou et al. [31]. We then converted the methylation averages to M-values (Mi = log2(Betai/(1-Betai)) [32], and fit M-values to a 3-component Gaussian Mixture Model (GMM) using the mclust R package [33]. Based on its mean, we assigned the three components labels of low, intermediate, and high. Each bin was labeled as a PMD if the probability of being in the high bin was less than 0.01, and multiple consecutive PMD bins were merged into a single PMD call. Common ovarian cancer PMDs (ovcaPMDs) were defined as regions identified from PMDetect in more than 9 of our samples. Common ovca PMDs were combined with PMDs from other cancer cell types [31]. Solo-WCGW scores were calculated by averaging the methylation of solo-WGCW CpGs (using the definition from Zhou et al. [31]) within the combined ovca + commonPMD set. Taken together these PMDs spanned 69.57% of the genome, comprising 14.96% of the genome spanned by ovcaPMDs and 54.6% of the genome spanned by the common PMD set.

#### Identifying variable CpGs that overlap known mQTLS

Methylation quantitative trait loci (mQTL) from two publicly available datasets that represent known mQTLs in normal tissues [34] and cancer [35]. mQTLs with *P*-value < 5.0 × 10^–8^ were included for intersection with the 10,000 most variable CpGs across the cohort after PMD masking. This included 4,045,382 unique CpGs that are mQTLs across normal tissues and 41,057 unique CpGs that are mQTLs across 23 cancer types.

#### Calling differentially methylated regions (DMRs)

PMDs were masked from each sample’s BED file before conducting differentially methylated region (DMR) analysis. The Bioconductor package dmrseq [36] was utilized to identify DMRs between *BRCA1/2* carriers and *BRCA1/2* non-carriers using default settings. Metilene [37] was used to identify DMRs between matched primary and recurrent tumors from each patient using the following parameters: -M 500, -m 5, p 0.1, -c 5. Only DMRs that overlapped between two or more patients were retained. Bedtools [38] merge function was performed on all overlapping DMRs to merge regions within 250 bp using parameter -d 250. Heatmaps were plotted using mean methylation across each identified DMR. Enrichment analysis of DMRs was conducted using annotatr [39] and ChIPseeker [40]. We also included an enrichment analysis of several other genomic classifications, such as DNA methylation valleys, and PRC2 binding regions. In the package annotatR, we searched for enrichment in coding regions, intergenic regions, exons, introns, 3UTR, CpG shores, shelves and islands. Using ChIPseeker, we searched for enrichment in the default regions: promoter, 5’UTR, 3’UTR, exons, introns, and intergenic regions. We refined promoter regions based on distance from transcription start site (TSS) and redefined CpG islands and CpG shores as non-TSS or TSS if the region was within 2kB of a TSS. Backgrounds for enrichment controlled for either DMR size only or DMR size plus CpG count. A DMR size only background was generated using bedtools shuffle [38, 41]. DMR size plus CpG count background was generated using an in-house developed script.

#### Replication of DMRs with publicly available datasets

Genome-wide methylation data for primary HGSOC tumors from 20 *BRCA1/2* carriers and 60 *BRCA1/2* non-carriers from the Illumina 450 k array was downloaded from the Gene Expression Omnibus (GEO) at Accession GSE65821 [7]. DMR calling was performed using minfi [42], which requires two probes within 300 bp to have > 20% change in beta value in the same direction. Due to the promoter-focused design of the array only 31 of our 135 DMRs identified in the comparison of *BRCA1/2* carriers and non-carriers intersected two or more 450 k probes within 300 bp. Many of our DMRs were within enhancers that were intergenic or in intron 1. To include local regions (primarily promoters) with sufficient probe coverage such that minfi could identify DMRs should they be present, we extended the boundary of each of the 135 DMRs by 1 kb. 70 of our 135 DMRs now met the basic criteria for minfi to identify a DMR at that location, should one exist. DMR analysis of all probes within this set of coordinates did not produce any replication.

#### RNA-Seq quantification and statistical analysis

Reads within each fastq file were first trimmed using TrimGalore to remove low quality bases and sample barcodes, retaining reads 75 bp or longer. Each transcriptome is then aligned to hg38 and the Gencodev29 primary assembly [41]. Genes are quantified with RSEM [43] and Kallisto [44]. Sample-specific gene models were generated using alignments produced with STAR two pass mapping and Stringtie [45]. Gene expression values were shown as normalized variance stabilizing transformation (vst) counts. To measure RNA abundance, we first obtained BAM alignment quality metrics using Picard (http://broadinstitute.github.io/picard). Samples with less than 90 percent of reads mapped to the correct strand of the reference genome (PCT_CORRECT_STRAND_READS) were omitted. Patients whose primary and recurrent tumors both passed this quality control were retained (*n* = 50) (Additional File 1, Supplementary Table 2). Read counts were quantified using the R package Salmon [46] at transcript level and reads were mapped to Genecode Release 29 (GRCh38) comprehensive gene annotations by R package ‘tximport’ [47]. To filter out potential artifacts and very low expressed transcripts we retained transcripts with length greater than 300 bp, TPM (Transcripts Per Million) value greater than 0.05 and isoform percentage greater than 1%. Transcripts in blacklist regions [48] were also filtered out. We retained transcripts expressed in more than 5 samples, which resulted in 91,411 transcripts from 33,969 genes. Tumor purity was estimated by the degree of heterogeneity of the tumor microenvironment. We applied the R package ‘consensusTME’ [49] to estimate cell type specific enrichment scores based on TCGA ovarian cancer data, and confirmed there was low stromal content and generally low infiltration by immune cells across the samples (Additional File 2, Supplementary Fig. 1).

Differentially expressed genes between the *BRCA1/2* carrier versus *BRCA1/2* non-carrier and primary versus recurrent tumors were detected by R package ‘DESeq2’ [50]. The *P*-values were adjusted for multiple testing using the Benjamini–Hochberg procedure. Since our experiment design has group-specific effects, comparisons between *BRCA* carrier status are made between patients, while comparisons between primary versus recurrent tumors are made within the patient. To control for confounding differences between the primary and recurrent tumors from patients we constructed a nested DEseq2 model with formula; ~ purity + BRCAStatus + BRCAStatus:PatientID + BRCAStatus:isRecur, which has the main effect for BRCA status plus nested interactions with primary and recurrent status. To see whether the identified gene sets (e.g. genes inside PMDs or differentially expressed genes) show significant functional concordance, we performed Gene Set Enrichment Analysis [51] for Kyoto Encyclopedia of Genes and Genomes (KEGG) pathways [52]. We implemented enrichment analysis with R package ‘clusterProfiler’ [53]. For each enrichment analysis, we set the number of permutations to 10,000 and reported enriched pathways with a Benjamini–Hochberg adjusted *P*-value less than 0.05.

#### Linking enhancers to target genes

Correlation between DMRs and gene expression was performed by comparing primary and recurrent tumors from each individual patient, for samples with matched WGBS and RNA-seq available for both primary and recurrent tumors (*n* = 27). Only DMRs that overlapped between two or more patients were included in this analysis. Using GENCODE28, DMRs regions > 2 kb from any TSS were annotated as “distal” and regions < 2 kb from TSS were annotated as “promoter”. Distal regions were mapped to the closest genes (10 upstream and 10 downstream) and the promoter to the closest gene and the correlation between their expression and methylation measured, where average beta value of the DMR correlates (using Spearman test) to a change (positive or negative) in expression of the nearby genes. ELMER version 2.8.3 [54] was used to map the genes, and the correlation was performed to each link (DMR—gene) only using the samples in which the DMR was identified using the function cor.test in R. Links with a minimum P-value of 0.05 were retained.

## Results

### Whole genome bisulfite sequencing (WGBS) and transcriptomic profiling in matched primary and recurrent high grade serous ovarian cancer

**Fig. 1:**
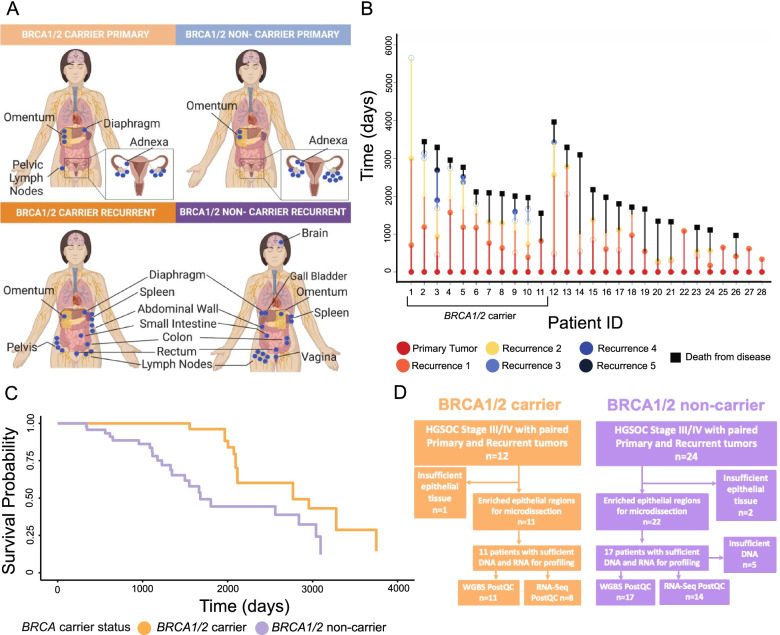
Clinical features of high grade serous ovarian cancer patients with recurrent disease: **A** Location of primary and recurrent tumors in women with and without germline *BRCA1/2* mutations; **B** Disease course for each patient. Filled circles indicate tumors that were profiled in this study, open circles tumors that were collected but not profiled; **C** Women carrying germline *BRCA1/2* mutations have an improved survival compared to non *BRCA1/2* mutation carriers; **D** Sample selection and QC process for inclusion in analysis

We used WGBS to perform whole genome methylation profiling and RNA sequencing (RNA-seq) for whole transcriptome profiling of 62 fresh frozen matched primary and recurrent tumor tissues from 28 women diagnosed with stage III/IV HGSOC. Clinical features of the patients and their tumors are given in Additional File 1, Supplementary Table 1 and illustrated in Fig. 1A and B. Eleven of these patients carried a pathogenic germline mutation in *BRCA1* and/or *BRCA2*. The remaining 17 patients were confirmed as non-*BRCA1/2* germline mutation carriers (Additional File 1, Supplementary Table 1). All patients received similar first line treatments comprising optimal debulking surgery followed by combination chemotherapy with a platinum agent. Time to first recurrence and median survival times were significantly greater in *BRCA1/2* carriers compared to *BRCA1/2* non-carriers (2768 days vs 1678 days respectively, *P*-value = 0.0056) (Fig. 1B and C).

For each primary and recurrent tumor specimen, we generated a bisulfite converted library and sequenced to a depth of ≥ 30x, generating approximately 400 million read pairs per library, with a bisulfite conversion rate greater than 99%. After removing CpGs covered by fewer than 5 reads, we obtained on average 24.2 million CpGs per tissue specimen (range 13.1—26.5 million), with an average of 24.6 million CpGs in primary tumors (15.9—26.5 million) and 23.8 million CpGs in recurrent tumors (13.1—26.4 million). RNA sequencing was performed on the same tissue samples. We generated a mean of 335 million reads from each library. Samples with less than 90 percent of reads mapped to the correct strand of the reference genome were excluded. After removing very low expressed transcripts (< 1 transcript per million (TPM)) and transcripts in ‘blacklist’ regions [48], we obtained 91,411 transcripts from 33,969 genes, coding and non-coding (Fig. 1D).

### Hypervariability in the landscape of partially methylated domains (PMDs) drives tumor heterogeneity between patients

**Fig. 2:**
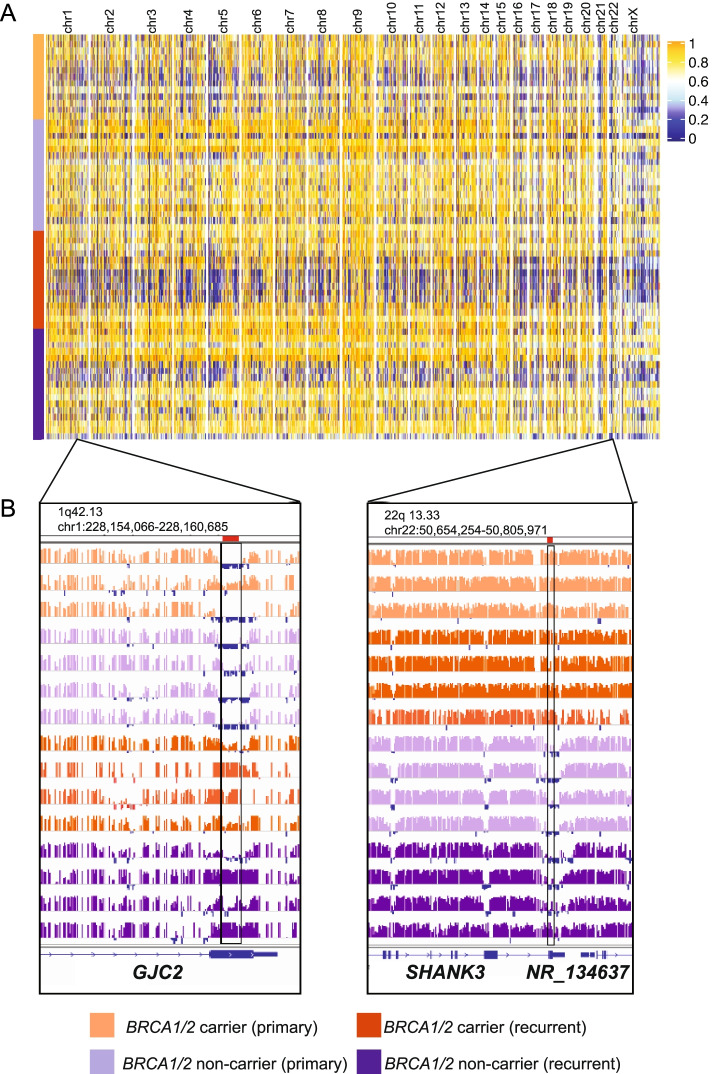
High grade serous ovarian cancers show heterogeneous patterns of genome-wide methylation: **A** Both primary and recurrent tumors show heterogeneous patterns of methylation across the genome, with many tumors showing extensive hypomethylation on the X chromosome (CpG values are averaged across 10kB windows, minus ENCODE ‘blacklist’ regions); **B** Examples of two regions on chromosome 1q42.13 and 22q13.33, that show differentially methylated regions (boxed regions) from two comparisons—Primary vs Recurrent tumors (left) and *BRCA1/2* carrier vs *BRCA1/2* non-carrier (right)

Genome-wide maps of all CpG sites using supervised (Fig. 2A) and unsupervised analysis (Additional File 2, Supplementary Fig. 2) indicated widespread heterogeneity between tumors from different patients. Unsupervised clustering in each of the four sample groups (primary, recurrent, *BRCA1/2* carrier and *BRCA1/2* non-carrier) showed that methylation profiles in primary and recurrent tumors consistently clustered according to patient status rather than their sample group classifier, except that methylation profiles also clustered by *BRCA1/2* mutation status (Additional File 2, Supplementary Fig. 3A-D).

**Fig. 3:**
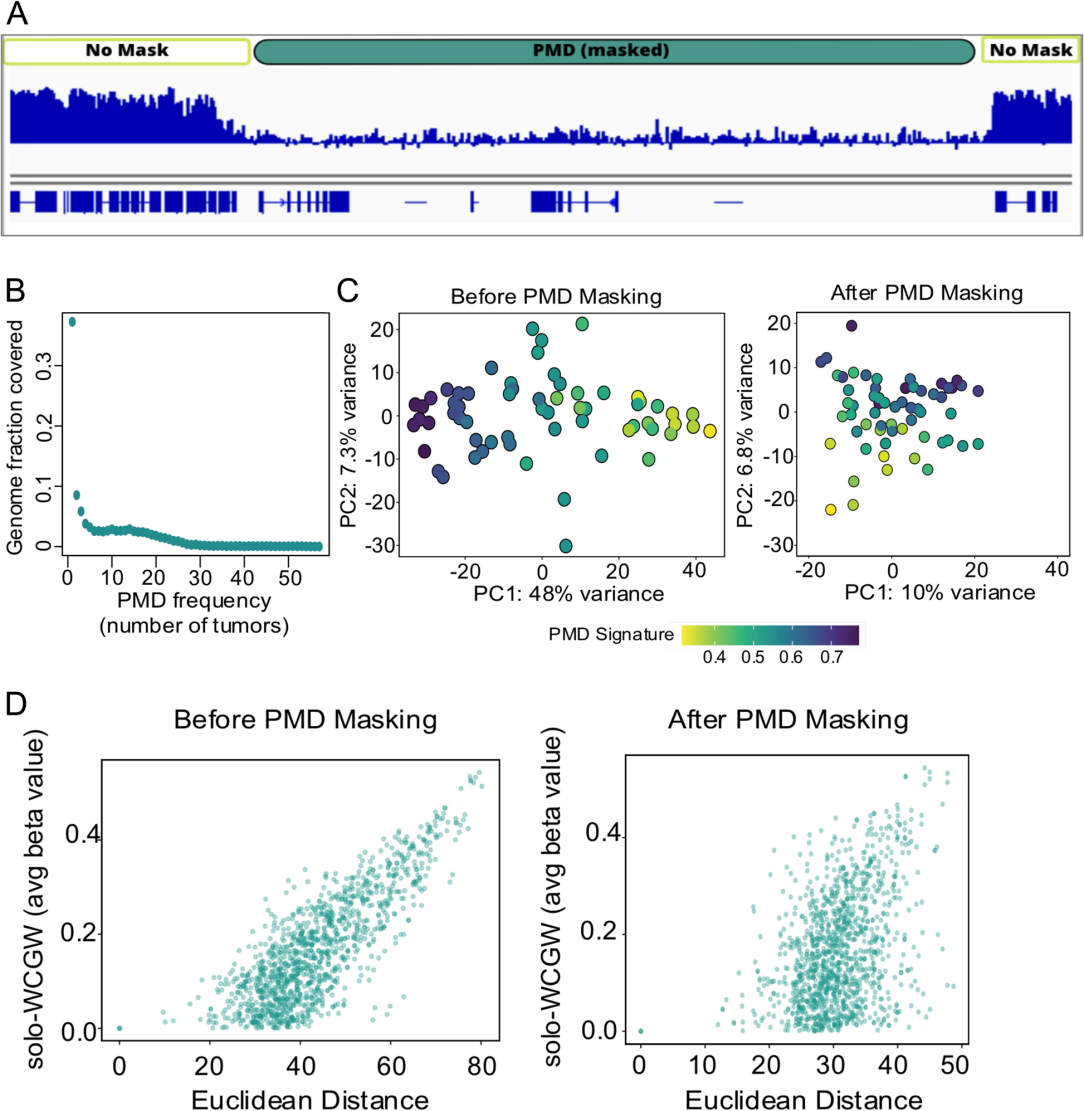
Hypermethylation within partially methylated domains (PMDs) in high grade serous ovarian cancers is driven by soloWCGWs: **A** Illustration of PMD-masking strategy prior to calling differentially methylated regions. PMDs were identified as described in methods, and then those genomic regions were masked out of subsequent analyses. **B** Most PMDs detected across the cohort were unique to a single tumor, with only 2% of PMDs observed in more than 30 tumors; **C** Principal components (PC) analysis identifies a large proportion of the variance between tumors was due to methylation at soloWCGW sites within PMDs. Masking the genome for common PMDs and ovcaPMDs removed much of the variance; **D** Pairwise comparison for all possible tumor pairs. The strong correlation between PMD soloWCGW difference and pairwise Euclidean distance was lost after masking PMDs

We attributed this heterogeneity to a global loss of methylation within large genomic blocks (partially methylated domains or PMDs) (Fig. 3A) [18, 31, 55]. The fraction of the genome covered by PMDs varied from 1–58% in this set of tumors, with an average of 29% of each tumor the genome covered by PMDs (Additional File 1, Supplementary Table 3). There was no consistent pattern in the PMD architecture across this series of tumors. The most frequently observed PMDs were detected in 56/62 tumors representing less than 0.03% of PMDs characterized in this set of tumors (Fig. 3B). We identified a set of ‘common’ PMDs that were shared in more than 9 tumor specimens (ovcaPMDs), determined by the first inflection point of the bimodal distribution seen by plotting PMD frequency (Fig. 3B; Additional File 1, Supplementary Table 4). Principal Component (PC) analysis using the top 10,000 most variable CpGs genome-wide (Fig. 3C, left) showed that PC1 accounted for 48% of the total variance.

Hypervariable methylation patterns due to PMDs can be reproduced using only methylation values from soloWCGW CpGs, which are flanked by an A or T on both sides (palindromic) and reside alone within a window of ± 35 bp [18, 31]. Thus, the average soloWCGW CpG methylation was calculated to represent the PMD signature within a maximal set of PMDs that included both cell-type ‘invariant’ common PMDs [31] and ovcaPMDs. PC1 was highly correlated with soloWCGW PMD methylation (Pearson correlation = 0.74, *P*-value = 4.5 × 10^–12^, Fig. 3C, left). When we removed CpGs located within common + ovcaPMDs and re-performed PC analysis the variance accounted for by PC1 was reduced from 48 to 10%, and the overall association with soloWCGW PMD methylation was greatly reduced (Pearson correlation = 0.31, *P*-value = 0.01, Fig. 3C, right), demonstrating that methylation in PMD regions contributes significantly to the heterogeneity across these tumors.

We also examined the effects of PMD masking by performing pairwise comparisons of soloWCGW methylation difference and Euclidean distance (calculated based on the 10,000 most variable CpGs) between each tumor (Fig. 3D). The correlation between delta soloWCGW and pairwise Euclidean distance was significantly attenuated after PMD masking (Pearson correlation = 0.65 and Pearson correlation = 0.17, respectively), reinforcing the observation that hypervariability at PMDs across the cohort contributes significantly to the observed heterogeneity.

**Fig. 4:**
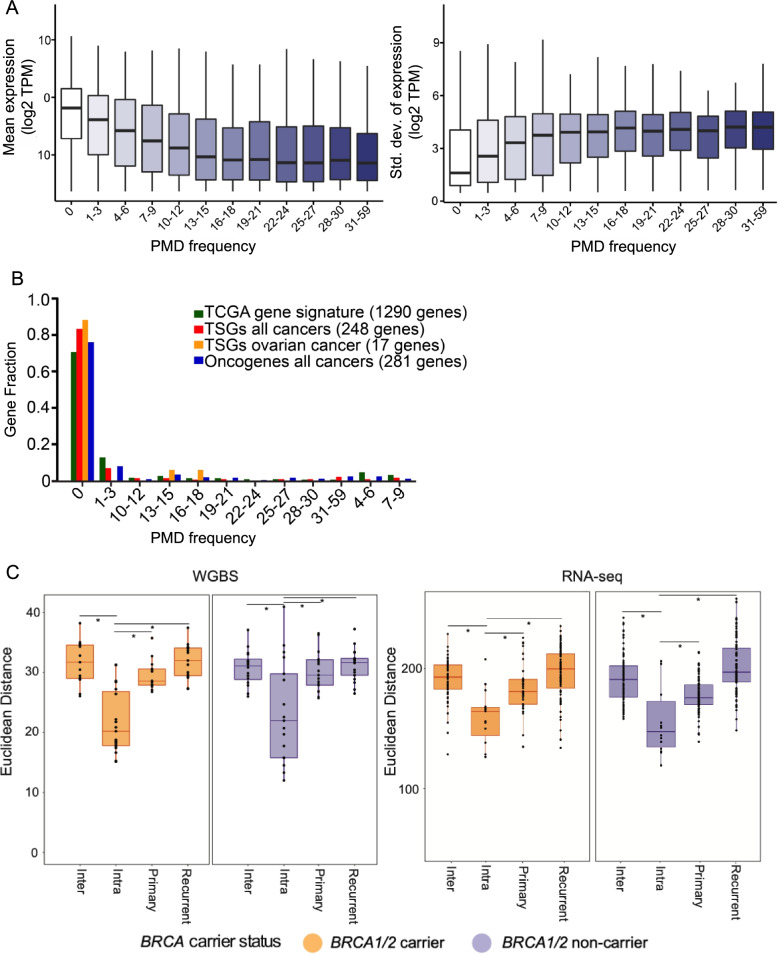
Methylation and transcription are largely preserved between primary and recurrent tumors from each patient, as shown by the expression of genes within partially methylated domains (PMDs): **A** Genes within PMDs shared by multiple tumor specimens are less expressed (left) but more variable in their expression (right) than genes outside of PMDs; **B** The vast majority of tumor suppressor genes in cancer and genes that form the ovarian cancer molecular subtypes defined by The Cancer Genome Atlas are located outside of PMDs; **C** Intra-patient pairwise Euclidean distances were significantly smaller than inter-patient distance or the intra-stage stage distance in both methylation (top) and gene expression (below) from paired RNA-Seq

CpG islands (CGIs) located within PMDs were hypermethylated compared to CGIs outside PMDs (Additional File 2, Supplementary Fig. 4A). We extended this analysis to include other functional genomic elements. We observed increased levels of methylation at promoters but not CGI shores, gene bodies or intergenic regions within PMDs (Additional File 2, Supplementary Fig. 4B). Whole transcriptomic profiling in the same tumor specimens was used to evaluate correlations for genes within common + ovcaPMDs. Genes within these PMDs were expressed at a significantly lower level than genes outside PMDs, with the lowest expression for genes inside the most common PMDs (*P*-value < 2 × 10^–16^, linear regression; Fig. 4A, left panel). Also, there was more variability in the expression of genes within PMDs than for genes outside PMDs (*P*-value < 2 × 10^–16^, linear regression; Fig. 4A, right panel). These findings were similar in tumors from different sample groups (*BRCA1/2* carrier and non-carriers, and from primary and recurrent tumors) (Additional File 2, Supplementary Fig. 5A, B) and consistent with previously reported relationships between methylation and gene expression in PMDs [18].

We next examined the frequency of known or predicted tumor suppressor genes (TSGs) [56] located in common + ovcaPMDs. Seventeen percent of known TSGs (41/248) were located in these PMDs, of which only 17 TSGs (7%) are located in low-frequency PMDs (hypergeometric test *P*-value = 4.17 × 10^–17^; Fig. 4B). TSGs known to be associated with ovarian cancer development were excluded from PMDs (*P*-value = 0.002). Seventy-one percent of the cancer genome atlas project (TCGA) signature genes (*P*-value = 2.75 × 10^–18^) that define the four molecular subtypes of HGSOC [8] and 73% of pan-cancer oncogenes [56] were located outside PMDs (*P*-value = 1.94 × 10^–9^; Fig. 4B). Taken together, these findings suggest that PMDs are depleted at genes important in cell identity and function. Gene Set Enrichment Analysis (GSEA) for genes located within PMDs shows that these genes are enriched in the Rig-I-like receptor signaling and ERBB signaling pathways (Additional File 1, Supplementary Table 5), similar to previously reported findings in breast cancer [18] and human neuron cells [57].

We masked the genome for PMD methylation to remove the influence of highly variable PMDs and then reanalyzed the data. Hierarchical clustering and PC analysis for the 10,000 most variable CpGs showed that recurrent tumors continued to cluster most closely with the matched primary tumor from the same patient rather than by primary, recurrent or *BRCA1/2* mutation status (Additional File 2, Supplementary Fig. 6A, 5B). None of the 10,000 most variable CpGs are known methylation QTLs in normal or cancer tissues, indicating this clustering was not driven by germline genetic variation [34, 35]. To quantify these clusters we calculated the Euclidean distances from each primary and recurrent tumor pair from the same patient (intra-patient distances) and the distances of all pairs from different patients (inter-patient distances). Intra-patient distances were significantly smaller than inter-patient differences in both *BRCA1/2* carriers and non-carriers (*P*-values = 7.16 × 10^–7^ and 1.41 × 10^–3^ respectively; Fig. 4C). For further validation of this finding, we evaluated heterogeneity associated with whole transcriptome data. Similarly, intra-patient Euclidean distances were significantly shorter than inter-patient distances in both *BRCA1/2* carriers and non-carriers (*P*-values = 9.67 × 10^–5^ and 6.70 × 10^–4^ respectively; Fig. 4C). These observations are unlikely to be the result of residual PMD hypomethylation differences after masking, but more the epigenetic landscape of recurrent tumors defined by inter-patient heterogeneity.

### HGSOCs maintain methylation and gene expression programs in primary tumors progressing to chemoresistance

We used metilene to perform paired analysis of the primary and recurrent tumors from each patient [37] to identify changes in methylation associated with recurrence after a diagnosis of primary HGSOC. We identified 15,082 DMRs with > 10% change in methylation and Q < 0.1 across all primary and recurrent tumor pairs, but found no DMRs meeting a genome-wide corrected significance threshold of Q < 0.05. We found significantly more DMRs (> 10% change, Q < 0.1) in primary versus recurrent tumors from *BRCA1/2* non-carriers (11,205 DMRs in 16 tumor pairs, average of 659 DMRs per case comparison) compared to tumors from *BRCA1/2* carriers (3,877 significant DMRs in 11 tumor pairs, average of 388 DMRs per case comparison) (*P*-value = 0.004). We restricted these analyses to 1,785 DMRs that were shared between primary and recurrent tumors from 2 or more patients (Additional File 1, Supplementary Table 6). We calculated the change in methylation at each CpG site within these DMRs between primary and recurrent tumors from each patient. Of these DMRs, 558/1,785 (31%) were discordantly methylated; in other words, the same region is observed as hyper- and hypo-methylated in different patients (Additional File 2, Supplementary Fig. 7). Hierarchical clustering analysis failed to identify any clinical or molecular features that correlate with consistent methylation changes at these regions. Taken together, these data indicate that the methylation landscapes of recurrent HGSOCs are largely conserved after chemotherapy and disease recurrence, and do not acquire common somatic methylation changes that may be drivers of chemoresistance and tumor recurrence.

Whole transcriptome data identified 99 genes that were differentially expressed between all primary and recurrent tumors (adjusted P-value < 0.05), of which 37 genes had lower expression and 62 genes higher expression in recurrent compared to primary tumors (Additional File 1, Supplementary Table 7). Differential expression for twenty of these genes was directionally consistent with differential changes in methylation (Additional File 1, Supplementary Table 8). We compared the DEGs we identified with 1,836 known/characterized tumor suppressor genes and oncogenes associated with pan-cancer development and molecular signature genes for different HGSOC subtypes described by TCGA [8]. The oncogenes *CCNE1* and *DDX5* were differentially expressed between primary and recurrent tumors. *CCNE1*, which is significantly amplified and highly expressed in aggressive HGSOC cases with poor clinical outcome [58], was overexpressed in recurrent tumors (*P* = 3.65 × 10^–6^). *DDX5* was overexpressed in recurrent tumors and located within 10 genes of a DMR that is hypomethylated, although at ~ 270 kb from the gene (Additional File 1, Supplementary Table 8).

### Hypomethylation increases expression of immune-related genes and identifies putative drivers of disease recurrence in HGSOCs from *BRCA1/2* carriers

**Fig. 5:**
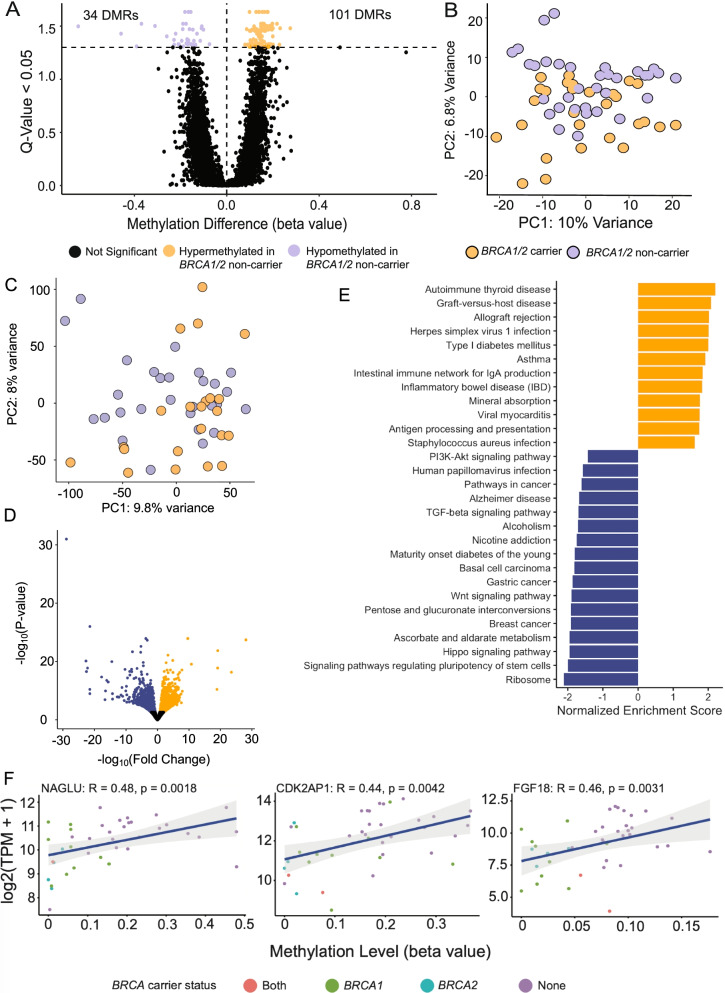
Significant differences in methylation and expression by *BRCA1/2* mutation carrier status: **A** 135 differentially methylated regions (DMRs) were identified in tumors from *BRCA1/2* carriers compared to non-carriers with a trend towards hypermethylation in tumors from non-carrier; 101 regions hypermethylated in tumors from *BRCA1/2* non-carriers compared to only 34 regions hypermethylated tumors from mutation carriers; **B** Principal components analysis using genome-wide CpG methylation level after PMD masking shows a trend towards differences in tumors based on *BRCA1/2* carrier status; **C** Principal components analysis of gene expression data shows a trend towards clustering of tumors based on *BRCA1/2* carrier status **D** Volcano plot of differentially expressed genes comparing tumors from *BRCA1/2* carriers vs non-carriers. Significantly up-regulated genes in tumors from *BRCA1/2* carriers are colored orange (Padj < 0.05), significantly down-regulated genes are colored purple; **E** KEGG gene set enrichment analysis for up-(orange) and down-(purple) differentially expressed genes in tumors from *BRCA1/2* carriers vs non-carriers; **F** Individual genes where methylation levels within hypermethylated regions in tumors from *BRCA1/2* non-carriers were correlated with gene expression (P-value < 0.005); *NAGLU*, *CDK2AP1*, *FGF18*

We identified 135 significant DMRs in primary-recurrent HGSOCs between *BRCA1/2* carriers and non-carriers (Q-value < 0.05) (Fig. 5A; Additional File 1, Supplementary Table 9), with a trend for hypermethylation at these DMRs in tumors from *BRCA1/2* non-carriers and genome-wide hypermethylation in PMD regions in primary tumors from *BRCA1/2* non-carriers (*P*-value = 0.0011) (Additional File 2, Supplementary Fig. 8). PCA analysis also suggested a trend for tumors to cluster together based on *BRCA1/2* carrier status (Fig. 5B). Annotation of DMRs hypermethylated in *BRCA1/2* non-carrier tumors showed these regions were depleted in CpG islands near transcription start sites and active regulatory regions marked by H3K27ac in the secretory epithelial fallopian tube cell line FT246 (a model for the precursor cell type for HGSOC) indicated by at least a 15% reduction in methylation over background (Additional File 2, Supplementary Fig. 9). This depletion indicates that while tumors from *BRCA1/2* carriers and non carriers are heterogenous, differential methylation between the two groups is focused at active regulatory regions in relevant cell types and CpG islands near transcription start sites, which play an important role in the regulation of gene expression.

We performed a replication analysis of the 135 DMRs using previously published array-based differential probe methylation data generated using Illumina 450 k array analysis of tumors from 20 *BRCA1/2* carriers and 60 non-carriers [7] (q < 0.05, Additional File 1, Supplementary Table 10). Masking probe locations that overlapped common + ovca PMDs reduced the total probe count on the array to 310,968 probes. These probes were intersected with our DMRs, and overlapping probes were used to identify DMRs between *BRCA1/2* carriers and non-carriers using the tool bumphunter [42], which requires two probes within 300 bp to have a significant change in methylation in the same direction of one another to be considered a DMR candidate region. After removing probes that did not overlap DMRs, replication data were available for 31/135 DMRs. We did not identify significant differentially methylated regions using bumphunter at any of these 31 DMRs. A similar replication analysis was not possible using TCGA dataset (613 HGSOCs including 52 *BRCA1/2* and 561 *non-BRCA1/2* tumors) because these tumors were analyzed using the Illumina 27 K methylation array for which there is very low concordance with our WGBS data (one CpG probe, cg21557231).

PCA analysis based on differential gene expression (DEG) analysis also identified clusters associated with *BRCA1/2* mutation status (Fig. 5C). We identified 3,341 DEGs in tumors from *BRCA1/2* carriers compared to non-carriers (adjusted *P*-value < 0.05) (Additional File 1, Supplementary Table 11) of which 1,760 genes were up-regulated and 1,581 genes down-regulated (Fig. 5D). This is consistent with DMR analysis where we found a greater proportion of hypomethylated CpGs in tumors from *BRCA1/2* carriers compared to non-carriers. Up-regulated genes in *BRCA1/2* associated tumors were significantly enriched in immune related pathways (Q < 0.05) including autoimmune diseases, infection response and antigen processing and presentation (Fig. 5E) even though there were no clear differences in immune cell infiltration in *BRCA1/2* tumors compared to non-*BRCA1/2* tumors (consesusTME scores *P*-value = 0.97) (Additional File 2, Supplementary Fig. 1). Down-regulated genes in tumors from *BRCA1/2* carriers were most significantly enriched in pathways that maintain stemness and cell differentiation, including the hippo signaling pathway (adjusted *P*-value < 0.05) (Fig. 5E).

We connected changes in the methylation of regulatory elements to changes in gene expression. One thousand seven hundred and eighty two genes were located within 10 genes up and down-stream of the 135 DMRs (Additional File 1, Supplementary Table 12) including 94 gene promoters that were within 2 kb of a DMR. We compared this list with the 3,341 DEGs identified in tumors of *BRCA1/2* carriers and non-carriers and identified 68 instances (including some duplicate genes) where DMRs showed either positive or inverse correlation between methylation and expression of the nearby gene (Additional File 1, Supplementary Table 12). This corresponded to 37 unique regions. These DMRs varied in length from 100 bp to 8 kb with the number of promoters within 2 kb of a single DMR ranging between one and six. Twenty-five unique genes were hypermethylated and down-regulated in *BRCA1/2* carriers while 41 unique genes hypomethylated and up-regulated in tumors from *BRCA1/2* carriers. (Additional File 1, Supplementary Table 12). We adapted the software tool ELMER [54, 59] to correlate methylation values in DMRs with the expression of nearby genes, identifying three genes—*FGF18*, *CDK2AP1*, and *NAGLU*—where methylation and expression were positively correlated (q < 0.05, r = 0.46, 0.44, and 0.48 respectively) (Fig. 5F). Positive correlation between methylation and expression can indicate that a gene’s function is affected by gene body methylation or possibly methylation of a nearby insulator [60], although paradoxically increased expression may also arise from a fully methylated promoter [61].

## Discussion

Whole genome bisulfite sequencing (WGBS) catalogs a comprehensive survey of the methylation level of CpG residues across the genome, with coverage of CpGs at several orders of magnitude greater than the array-based approaches that have largely been used up until now to characterize the genome-wide methylation status of tumors. In this study, WGBS analysis reported on the methylation status of, on average, 24.6 million CpG sites per tumor analyzed. This contrasts with methylation arrays that interrogate highly selected CpG sites, of which the most commonly used have been the Illumina 27 K, 450 K, and EPIC (850 K) arrays that evaluate 0.1%, 1.5% and 3.0% of CpGs in the genome, respectively.

To our knowledge, the current study is the first to use WGBS instead of an array to comprehensively map CpG methylation and the transcriptome in matched primary HGSOCs and tumor recurrences arising post chemotherapy in the same patient, and the first study to comprehensively map PMDs genome-wide in HGSOCs using WGBS. The data are consistent with studies of the PMD architecture of other cancers and tissues [13, 17, 18, 31, 55]. We identified a common set of PMDs, encompassing 15% of the genome that appear to show specificity to HGSOC. Within these PMDs, CpG islands and other functional genomic elements were highly methylated and genes were expressed at lower levels compared to those located outside PMDs [13, 17, 18]. While the distribution of PMDs in primary and recurrent tumors was heterogeneous, there was a highly statistically significant enrichment for genes involved in cancer development, and more specifically genes that are differentially expressed and have been used to stratify HGSOCs into different molecular subtypes, located outside PMDs. We postulate that the critical nature of these genes in both normal cell function and tumor development requires these genes to be active, both spatially and temporally in their differentiation into specific tumor phenotypes. PMD hypomethylation was a central feature of the variation in methylation we observed across samples. By masking these regions we were able to find differences in methylation between *BRCA1/2* carriers and non-carriers. Without PMD masking, these differences were not apparent.

Given that our study is the first WGBS analysis of primary HGSOCs and of their recurrences, replicating our results in an independent cohort presents significant challenges. The largest genomic studies of HGSOC has been performed by TCGA, which includes methylation analysis using the 27 K CpG array of 489 primary HGSOCs compared to 8 full thickness fallopian tube tissues, an analysis that identified 168 genes that were epigenetically silenced in HGSOC. Twenty nine of these genes showed significantly reduced RNA expression in non-*BRCA1/2* tumors analyzed in our study, including four of the fifteen top ranked genes (*AMT*, *LDHD*, *CFTR* and *BANK1*) that we identified in a comparison of gene expression in HGSOCs from *BRCA1/2* carriers compared non-carriers. Our WGBS analysis also identified some differentially methylated genes that have been identified in HGSOCs in other studies, notably *HOXA9* which was found to be methylated in up to 95% of ovarian cancers in a study of 80 primary tumors from Montavon and colleagues [62]. *MYO18B* inactivation has also been reported in chemoresistant HGSOCs; this gene was significantly downregulated in tumors from *BRCA1/2* carriers in our analysis [63]. Previous studies have shown that the global loss of 5-hydroxymethylcytosine (5hmC) in HGSOC occurs through disease progression and chemo-resistance [64]. Future analyses of other types of epigenetic regulation, including additional methylation modifications such as 5hmC of DNA and m6A methylation of RNA or histone modifications that include methylation and acetylation, may identify gene regulatory mechanisms that contribute to disease recurrence.

Previous studies have compared the molecular features of primary ovarian cancers (including methylation) with metastatic tumors or cells from ascitic fluid to identify molecular biomarkers and novel therapeutic targets for chemoresistant disease [7, 8, 65–67]. Generally, these studies suggest there is an accumulation of somatic mutations as tumors metastasize and recur after chemotherapy [7, 66], although no frequently somatically altered genes associated specifically with tumor metastasis/recurrence have yet been identified. While we did not perform whole genome sequencing of the same tumor specimen in the current study, our data from whole genome methylation and transcriptomic sequencing are largely consistent with previous findings. The most prevalent genes that were differentially altered in recurrent tumors were the known oncogene *DDX5,* which has been shown to be frequently amplified in breast cancers [68], and *CCNE1*, which is frequently amplified in HGSOC [58]. Otherwise, we found few molecular changes that were specific to tumor recurrence. Methylation and gene expression signatures were highly preserved in recurrences relative to the primary tumor from the same individual, but were significantly different between tumors from different individuals. There was little evidence of an accumulation of additional and novel methylation and transcriptomic changes in recurrent tumors that could be attributed to chemoresistance. This result is similar to a previous study of HGSOC using a methylation array that interrogated 91 patients with primary HGSOC and 6 non-matched patients with recurrent HGSOC [69]. This is notable as it indicates that the genomic changes required to promote chemo-resistance may be established early in the evolution of the primary tumor and persist to dominate the clonal populations of both the primary and subsequent recurrent tumors. This is consistent with a previous study of somatic mutations identified in multiple primary and metastatic samples from seven ovarian cancers, which found complex patterns of both monoclonal and polyclonal seeding of metastatic sites and predicted a lack of selective pressures after treatment with combination chemotherapy [70], and may indicate the presence of clones of chemoresistant disease at the time of primary tumor diagnosis. Thus, while the bulk of disease would respond to platinum-based therapies, a proportion of tumor cells may persist through chemotherapy to seed recurrent tumor growth within the peritoneal cavity.

Our WGBS analysis indicates that there are striking differences in the methylation profiles between patients with and without germline *BRCA1/2* mutations. The data suggest that hypermethylation is a feature of non-*BRCA1/2* associated tumors. This result was consistent within PMDs, specifically at soloWCGW CpGs and adds to the growing body of evidence that indicates non-*BRCA1/2* associated HGSOCs develop along different molecular pathways compared to HGSOCs from *BRCA1/2* carriers. Recent studies have suggested that foldback inversions may be drivers of HGSOC development in *BRCA1/2* non-carriers resulting in unique mutational processes that do not correlate with any of the different molecular subtypes described for HGSOC by TCGA [8, 23]. Our data support the findings of studies that have identified several notable genes that are significantly differentially expressed in non*-BRCA1/2* compared to *BRCA1/2* HGSOCs including: *EIF3CL* which regulates a cluster of metastasis-promoting genes via *STAT3* and acts as a mediator of immune cell evasion [71] and *CFTR* overexpression (also reported by TCGA) which is known to increase cell invasion, proliferation and adhesion in ovarian cancers [72], and is highly expressed in the fallopian tube secretory epithelial cells from which many HGSOCs arise [73]. In addition, we observed 48 non-coding transcripts where expression was significantly correlated to changes in methylation. 16/48 of these non-coding RNAs (ncRNAs)(AC125807.2, LINC00431, AP001626.1, LINC00858, LINC01234, AL021578.1, ARHGEF26-AS1, LINC-PINT, PAXIP1-AS2,PAXIP1-AS1, FIRRE, SPANXA2-OT1, FMR1-AS1, IRF1-AS1, STAM-AS1, MIR3936HG) are directly linked to several cancers, including ovarian cancer, esophageal cancer, gastric carcinoma, myeloma, non-small cell lung cancer, colon cancer, colorectal cancer, and hepatocellular carcinoma. ncRNAs can induce occurrence and progression of disease by working in concert with protein-coding genes to alter DNA repair in response to double strand breaks [74–78]. Cells without *BRCA* mutations are homologous recombination deficient, making it possible some of these ncRNAs are contributing to the differences we observe between *BRCA1/2* carriers and non-carriers. LINC01234, for instance, is differentially expressed in our cohort and has been shown to stratify non-carriers of *BRCA1/2* mutations patients based on survival, where high levels of expression are predictive of improved survival [79]. These ncRNAs may provide an avenue for potential therapeutic targets or prediction of chemosensitivity in HGSOC patients.

## Conclusions

Our study describes the first comprehensive analysis of paired methylation landscapes generated by WGBS in HGSOCs and their recurrences after chemotherapy, and the first such comparison in patients with and without germline *BRCA1* and *BRCA2* mutations. This study highlights the molecular heterogeneity that exists in HGSOCs between patients, consistent with single-cell analyses [80, 81], and provides evidence that this heterogeneity extends to chemoresistant, recurrent disease. We have shown that there is an absence of common methylation signatures or specific methylation biomarkers that would indicate common mechanisms and underlying biology associated with disease recurrence or chemoresistance. This observation was replicated using whole transcriptome profiling in the same primary-recurrent tissue specimens. Taken together, these data suggest that the methylation and transcriptomic changes required to survive first line chemotherapy and seed recurrent tumors may already be present in the primary tumor, rather than induced as a result of exposure to chemotherapy. The most significant methylation and/or transcriptomic variations were observed when we compared primary tumors with and without *BRCA1* or *BRCA2* mutations. The improved survival and disease free interval in patients with *BRCA1* or *BRCA2* mutations has been attributed to their improved response to platinum based chemotherapy, and we have identified extensive differences in the methylome and transcriptome between these groups that likely contribute to these differences.

## Supplementary Information

**Additional file 1:** **Supplementary Table 1.** Clinical features of the cohort. **Supplementary Table 2.** PostQC sample information. **Supplementary Table 3.** PMD genome coverage per sample. **Supplementary Table 4.** PMDs common to ovarian cancer (ovcaPMDs). **Supplementary Table 5.** Enriched pathways for genes in PMDs. **Supplementary Table 6.** DMRs from Primary vs Recurrent. Negative values indicate regions hypermethylated in recurrent samples. **Supplementary Table 7.** DEGs from Primary vs Recurrent. **Supplementary Table 8.** Genes near Primary vs Recurrent DMRs intersecting DEGs. **Supplementary Table 9.** DMRs from BC vs NC. **Supplementary Table 10.** DMRs from BC vs NC (hg19) that overlap 450k probes within 300bp. **Supplementary Table 11.** DEGs from BC vs NC. **Supplementary Table 12.** Genes near BC vs NC DMRs intersecting DEGs

**Additional file 2:** **Supplementary Figure 1.** Purity estimates using RNA-Seq data. ConsensesTME cell type estimations identifiedfour clusters of samples defined by a gradient of T cell, B cell Natural Killercell composition. ConsensusTME clusters did not correlate with BRCA1/2 mutationstatus, primary/recurrent status, RNA[1]Seq library quality,or clinical parameters. **Supplementary Figure 2.** Alternative presentation of heterogeneous genome-wide methylation patterns across cohort: Unsupervised clustering of genome wide CpG methylation level from primary and recurrent tumors shows heterogeneous patterns of methylation across the genome; CpG beta values are averaged across 10kB windows, minus ENCODE blacklist regions.  **Supplementary Figure 3.** Unsupervised clustering of 10,000 most variable CpGs in multiple comparisons. Unsupervised clustering of the tumors in each of the four sample groups: (A) primary, (B) recurrent, (C) BRCA1/2 non-carrier and (D) BRCA1/2 carrier. Tumors showed similar patterns of clustering by patient. Within primary tumors, BRCA1/2 carrier status also appeared to affect clustering. Tumors are annotated with primary and recurrent event information, promoter methylation at RAD51C and BRCA1 as an indicator of possible homologous recombination deficiency, batch and patient label (Case ID). CpG beta values shown on scale of 0-1. **Supplementary Figure 4.** HGSOC tumors show a high degree of heterogeneity within PMDs. (A) CGIs within PMDs are highly methylated, while those outside of PMDs are less methylated; (B) Functional elements in the genome are highly methylated when they fall within PMDs. **Supplementary Figure 5.** The expression and variability of genes within partially methylated domains (PMDs) in high grade serous ovarian cancer tumors: (A) Genes frequently located within PMDs are expressed at a lower level but are more variable in their expression than those rarely located in PMDs. This is observed in tumors from BRCA1/2 carriers and non-carriers (A) and in primary and recurrent tumors (B). **Supplementary Figure 6.** High grade serous ovarian cancers show a high degree of heterogeneity in methylation: (A) Clustering of the 10,000 most variable CpG sites in the genome after masking for partially methylated domains (PMDs) show that tumors do not cluster by BRCA carrier status or primary/recurrent tumor status, but by patient. Tumors are annotated with primary and recurrent event information, promoter methylation at RAD51C and BRCA1 as an indicator of possible homologous recombination deficiency, batch and patient label (Case ID); (B) PCA of 10,000 most variable CpGs after masking for PMDs shows there are no clear clusters of tumors based on primary vs recurrent status. In many patients, the primary and recurrent tumors cluster closely together, similar to the heatmap shown in (A). **Supplementary Figure 7.** Primary to recurrent tumor progression. Heatmap of differentially methylated regions (DMRs) from Primary vs Recurrent analysis, (plotted as the delta or change in methylation level between the primary and recurrent tumor) showed variable methylation in the same regions across our tumor sets. Other DMRs indicated relatively no change between primary and recurrent tumors (white regions on heatmap) indicating a stability in methylation profiles after chemotherapy. **Supplementary Figure 8.** Comparing tumors from BRCA1/2 carrier vs non-carriers: Tumors from BRCA1/2 non-carrier have significantly higher methylation at soloWCGW sites within partially methylated domains. **Supplementary Figure 9.** Enrichment of differentially methylated regions (DMRs) in tumors from BRCA1/2 carriers vs non-carriers: Enrichment of differentially methylated regions between tumors from BRCA1/2 carriers and non-carriers were compared to a background set of genomic regions, matching DMR length and CpG content. DMRs were considered enriched at regions where the percent change from background was >15%, indicated by dashed grey line. Regions hypermethylated in BRCA1/2 non-carriers were enriched in CpG islands associated with transcription start sites (CpG_TSS) and FT246 H3K27ac peaks. No enrichment was found for either hyper- or hypomethylated regions in 5’ untranslated regions (UTR), non-TSS CpG islands, non-TSS CpG shores, TSS CpG shores, DNA methylation valleys, Kuramochi H3K27ac peaks, PRC2 binding regions, or promoters 1-2kb upstream of TSS.

## Data Availability

This study did not generate any unique reagents or materials. Project data is available publicly at the Gene Expression Omnibus under accession number GSE202245. Code to generate most figures as well as unique code required for this study will be uploaded and maintained on Github by the first author, Nicole Gull: https://github.com/nicoleversetwo/methylation-foo

## Abbreviations

HGSOC: High grade serous ovarian cancer
DMRs: Differentially methylated regions
PMDs: Partially methylated domains
WGBS: Whole genome bisulfite sequencing
gDNA: Genomic DNA
GMM: Gaussian Mixture Model
ovcaPMDs: Ovarian cancer PMDs
mQTL: Methylation quantitative trait loci
TSS: Transcription start site
GEO: Gene Expression Omnibus
KEGG: Kyoto Encyclopedia of Genes and Genomes
TPM: Transcript per million
PC: Principal component
CGI: CpG island
TSG: Tumor suppressor genes
GSEA: Gene set enrichment analysis
DEG: Differential gene expression
