# Supplementary material for: DNA methylation and transcriptomic features are preserved throughout disease recurrence and chemoresistance in high grade serous ovarian cancers
Source: J Exp Clin Cancer Res. 2022 Jul 27;41:232. doi: 10.1186/s13046-022-02440-z (PMC9327231; doi:10.1186/s13046-022-02440-z)
Supplement: Supplementary file 2 — Additional file 2: Supplementary Figure 1. Purity estimates using RNA-Seq data. ConsensesTME cell type estimations identifiedfour clusters of samples defined by a gradient of T cell, B cell Natural Killercell composition. ConsensusTME clusters did not correlate with BRCA1/2 mutationstatus, primary/recurrent status, RNA[1]Seq library quality,or clinical parameters. Supplementary Figure 2. Alternative presentation of heterogeneous genome-wide methylation patterns across cohort: Unsupervised clustering of genome wide CpG methylation level from primary and recurrent tumors shows heterogeneous patterns of methylation across the genome; CpG beta values are averaged across 10kB windows, minus ENCODE blacklist regions. Supplementary Figure 3. Unsupervised clustering of 10,000 most variable CpGs in multiple comparisons. Unsupervised clustering of the tumors in each of the four sample groups: (A) primary, (B) recurrent, (C) BRCA1/2 non-carrier and (D) BRCA1/2 carrier. Tumors showed similar patterns of clustering by patient. Within primary tumors, BRCA1/2 carrier status also appeared to affect clustering. Tumors are annotated with primary and recurrent event information, promoter methylation at RAD51C and BRCA1 as an indicator of possible homologous recombination deficiency, batch and patient label (Case ID). CpG beta values shown on scale of 0-1. Supplementary Figure 4. HGSOC tumors show a high degree of heterogeneity within PMDs. (A) CGIs within PMDs are highly methylated, while those outside of PMDs are less methylated; (B) Functional elements in the genome are highly methylated when they fall within PMDs. Supplementary Figure 5. The expression and variability of genes within partially methylated domains (PMDs) in high grade serous ovarian cancer tumors: (A) Genes frequently located within PMDs are expressed at a lower level but are more variable in their expression than those rarely located in PMDs. This is observed in tumors from BRCA1/2 carriers and non-carriers (A) and [file 13046_2022_2440_MOESM2_ESM.pdf]

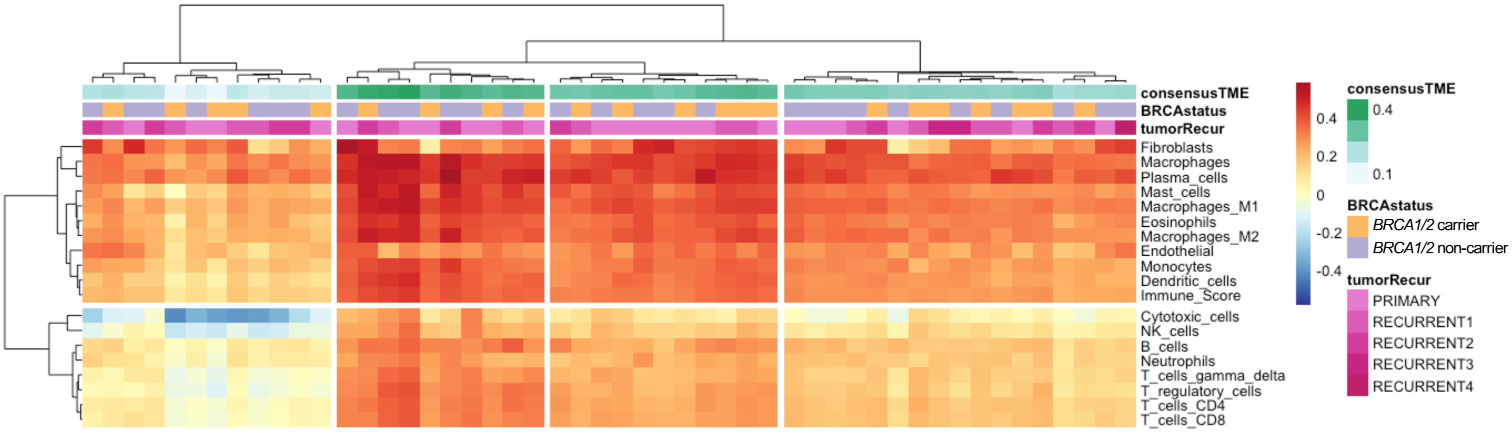

Supplementary Figure 1. Purity estimates using RNA-Seq data. ConsensesTME cell type estimations identified four clusters of samples defined by a gradient of T cell, B cell Natural Killer cell composition. ConsensusTME clusters did not correlate with *BRCA1/2* mutation status, primary/recurrent status, RNA-Seq library quality, or clinical parameters.

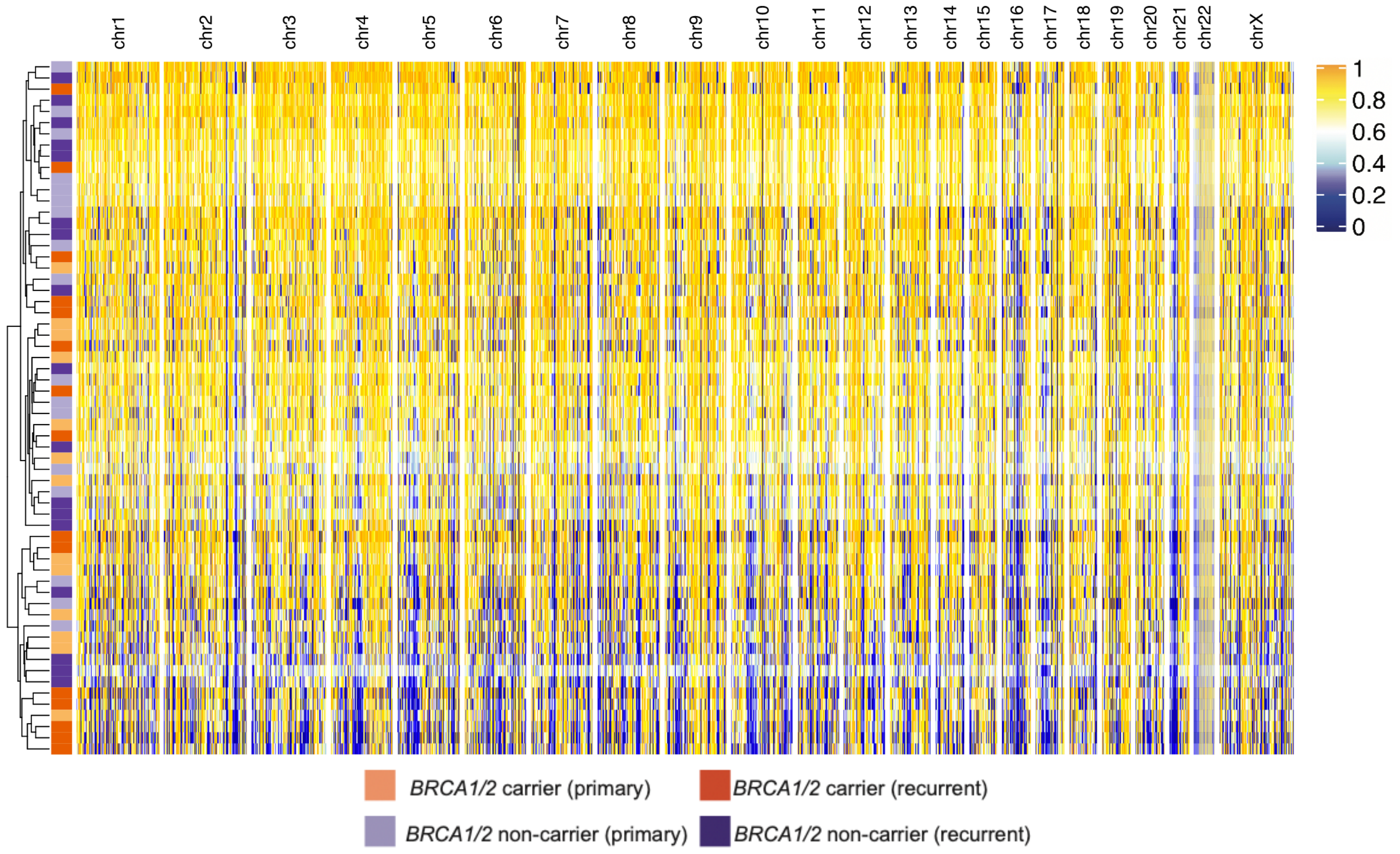

Supplementary Figure 2. Alternative presentation of heterogeneous genome-wide methylation patterns across cohort: Unsupervised clustering of genome wide CpG methylation level from primary and recurrent tumors shows heterogeneous patterns of methylation across the genome; CpG beta values are averaged across 10kB windows, minus ENCODE blacklist regions

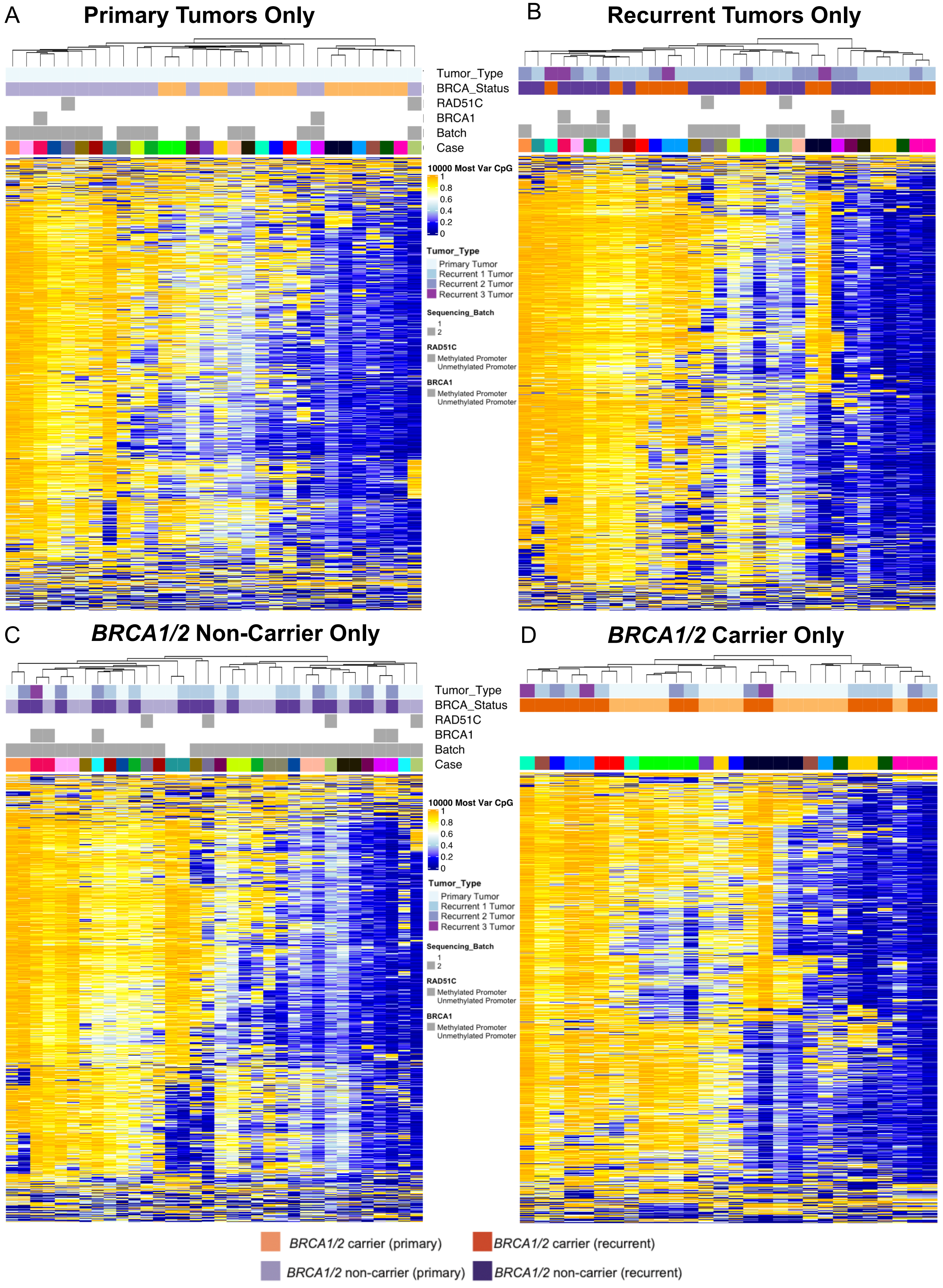

A
Primary Tumors Only
B
Recurrent Tumors Only
C
BRCA1/2 Non-Carrier Only
D
BRCA1/2 Carrier Only
Tumor_Type
BRCA_Status
RAD51C
BRCA1
Batch
Case
10000 Most Var CpG
1
0.8
0.6
0.4
0.2
0
Tumor_Type
Primary Tumor
Recurrent 1 Tumor
Recurrent 2 Tumor
Recurrent 3 Tumor
Sequencing_Batch
1
2
RAD51C
Methylated Promoter
Unmethylated Promoter
BRCA1
Methylated Promoter
Unmethylated Promoter
BRCA1/2 carrier (primary)
BRCA1/2 carrier (recurrent)
BRCA1/2 non-carrier (primary)
BRCA1/2 non-carrier (recurrent)

Supplementary Figure 3. Unsupervised clustering of 10,000 most variable CpGs in multiple comparisons. Unsupervised clustering of the tumors in each of the four sample groups: (**A**) primary, (**B**) recurrent, (**C**) *BRCA1/2 non*-carrier and (**D**) *BRCA1/2* carrier. Tumors showed similar patterns of clustering by patient. Within primary tumors, *BRCA1/2* carrier status also appeared to affect clustering. Tumors are annotated with primary and recurrent event information, promoter methylation at *RAD51C* and *BRCA1* as an indicator of possible homologous recombination deficiency, batch and patient label (Case ID). CpG beta values shown on scale of 0-1.

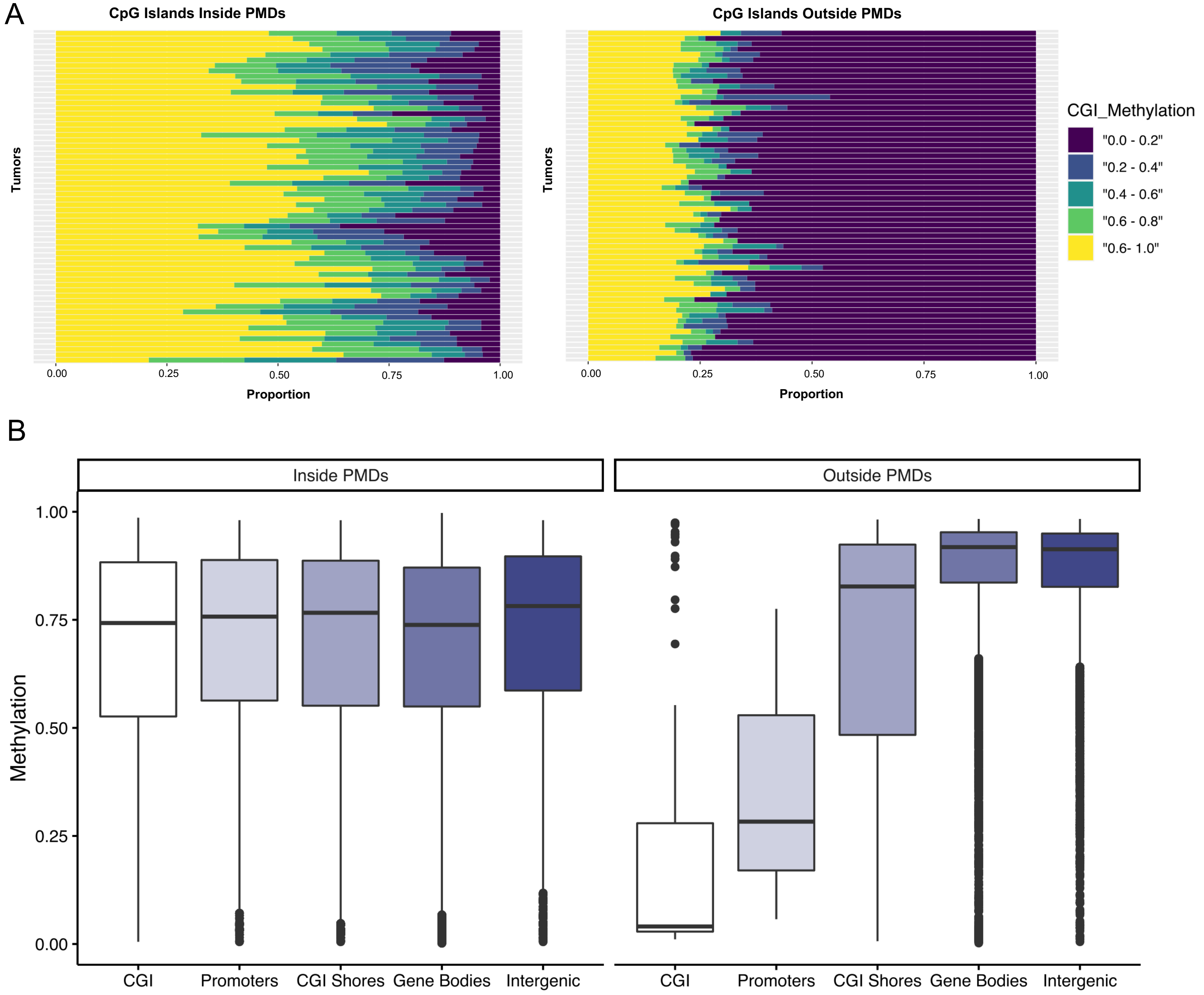

Supplementary Figure 4. HGSOC tumors show a high degree of heterogeneity within PMDs (A) CGIs within PMDs are highly methylated, while those outside of PMDs are less methylated; **(B)** Functional elements in the genome are highly methylated when they fall within PMDs.

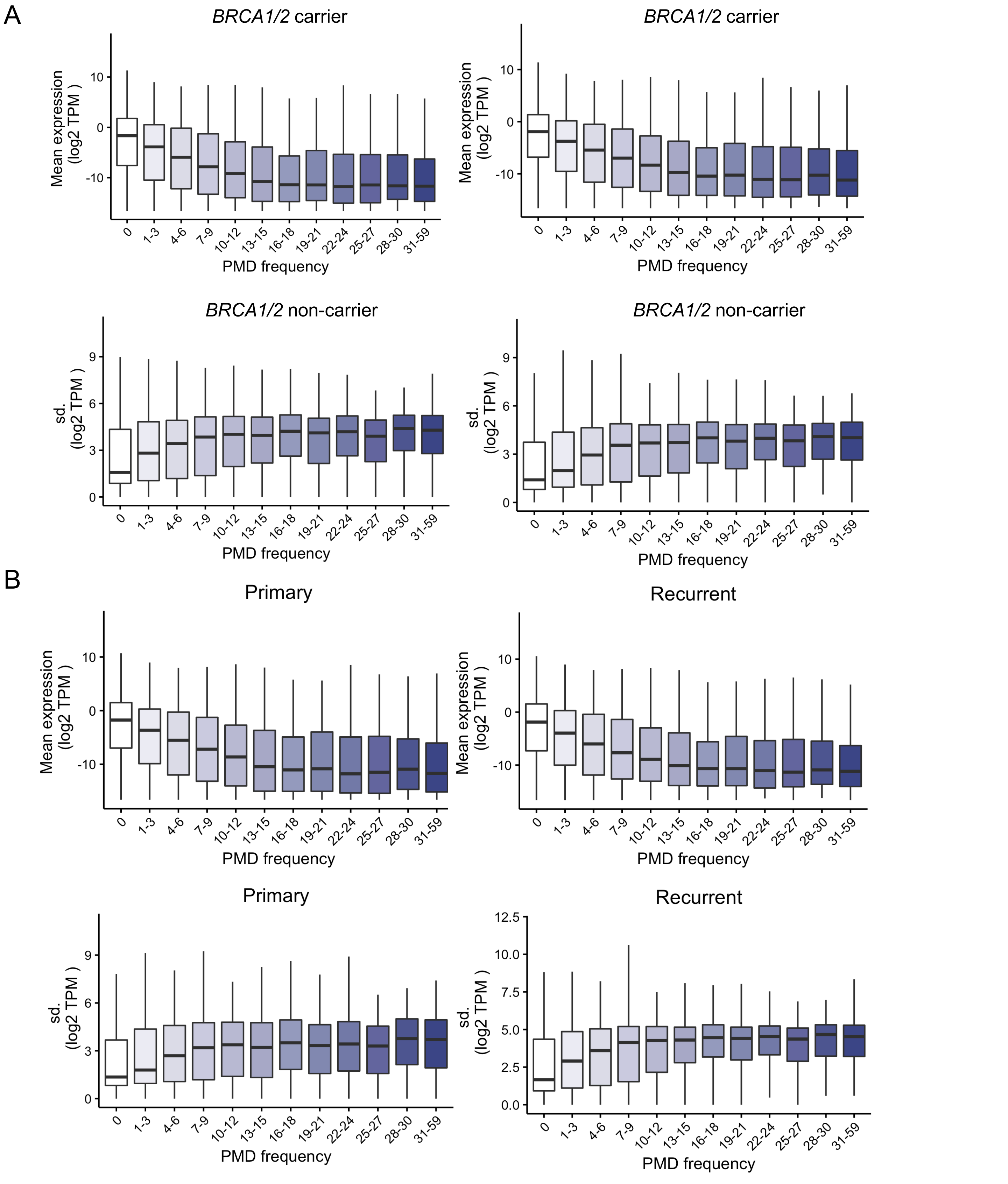

Supplementary Figure 5. The expression and variability of genes within partially methylated domains (PMDs) in high grade serous ovarian cancer tumors: (**A**) Genes frequently located within PMDs are expressed at a lower level but are more variable in their expression than those rarely located in PMDs. This is observed in tumors from *BRCA1/2* carriers and non-carriers **(A)** and in primary and recurrent tumors **(B)**.

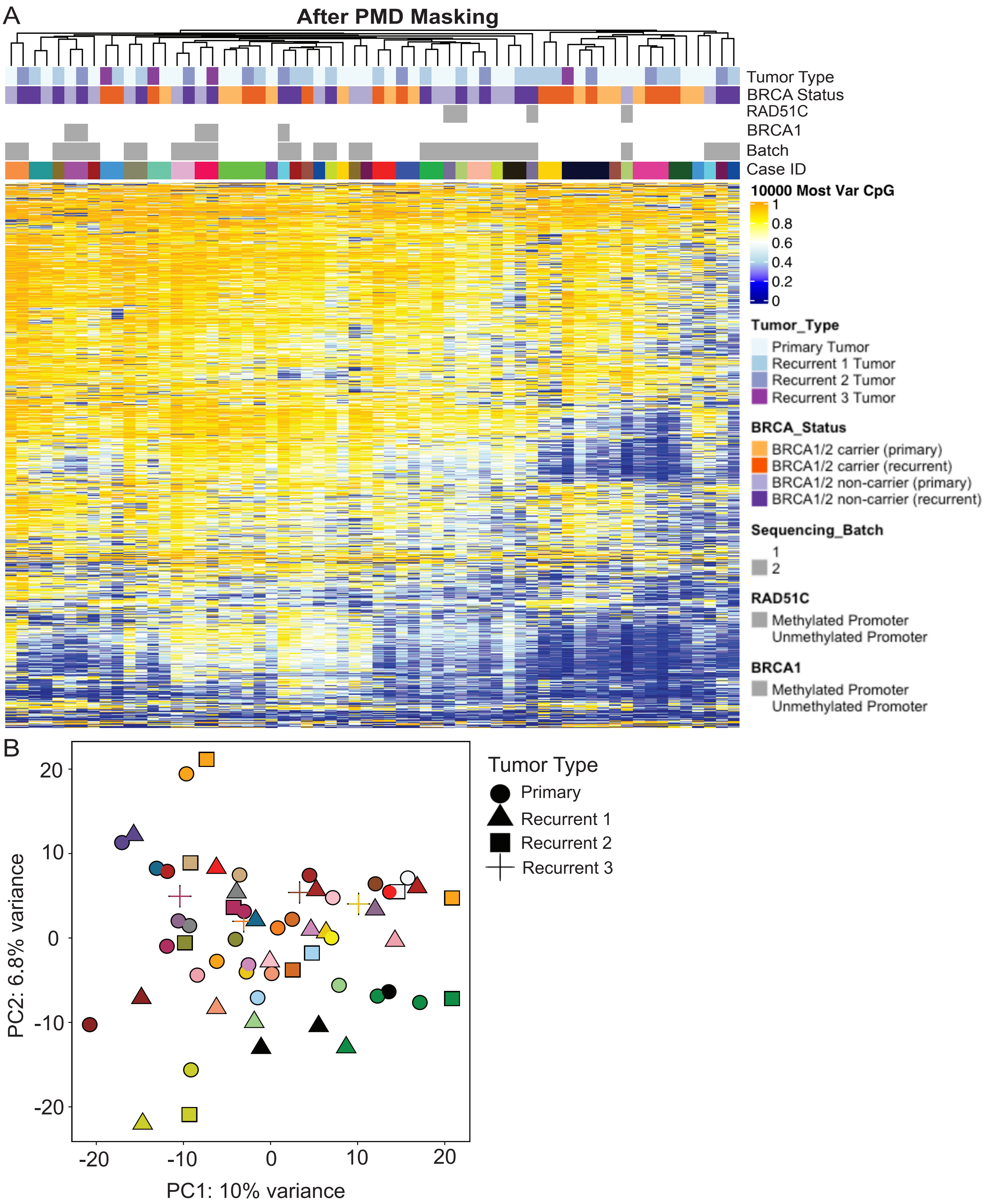

Supplementary Figure 6. High grade serous ovarian cancers show a high degree of heterogeneity in methylation: (A) Clustering of the 10,000 most variable CpG sites in the genome after masking for partially methylated domains **(**PMDs) show that tumors do not cluster by *BRCA* carrier status or primary/recurrent tumor status, but by patient. Tumors are annotated with primary and recurrent event information, promoter methylation at *RAD51C* and *BRCA1* as an indicator of possible homologous recombination deficiency, batch and patient label (Case ID); (**B**) PCA of 10,000 most variable CpGs after masking for PMDs shows there are no clear clusters of tumors based on primary vs recurrent status. In many patients, the primary and recurrent tumors cluster closely together, similar to the heatmap shown in (**A**).

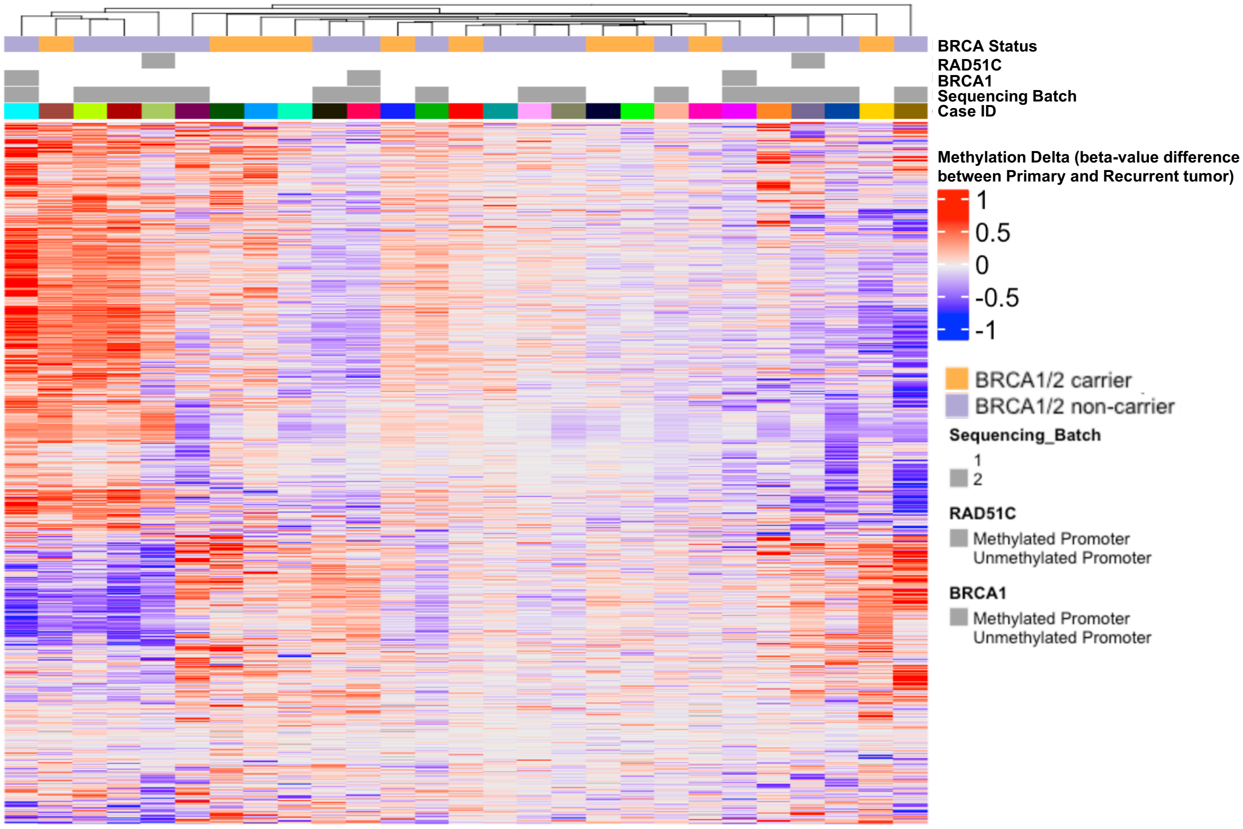

Supplementary Figure 7. Primary to recurrent tumor progression. Heatmap of differentially methylated regions (DMRs) from Primary vs Recurrent analysis, (plotted as the delta or change in methylation level between the primary and recurrent tumor) showed variable methylation in the same regions across our tumor sets. Other DMRs indicated relatively no change between primary and recurrent tumors (white regions on heatmap) indicating a stability in methylation profiles after chemotherapy.

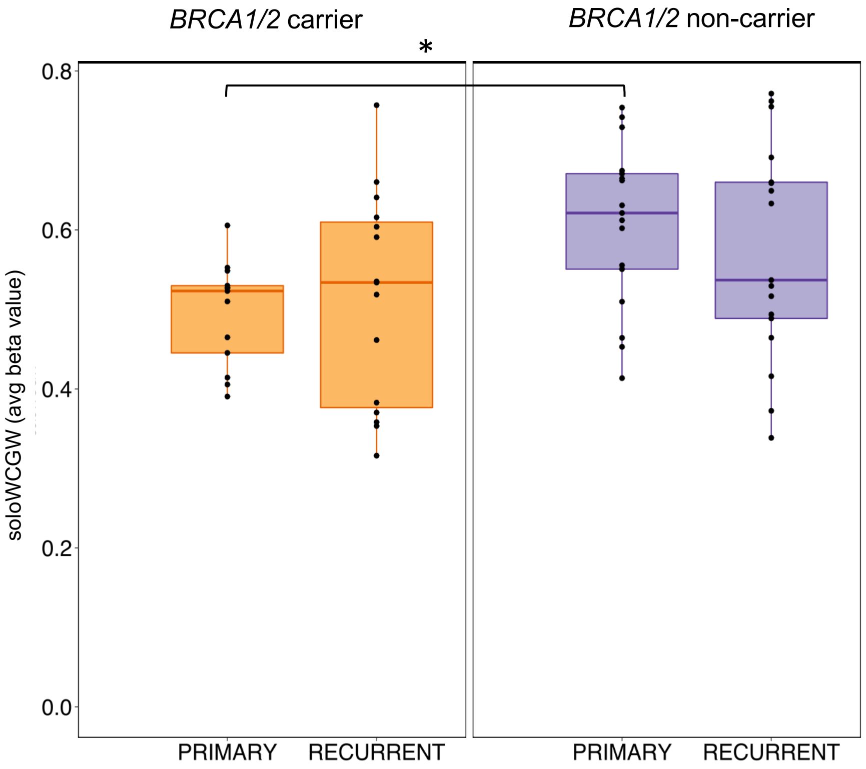

Supplementary Figure 8. Comparing tumors from *BRCA1/2* carrier vs non-carriers: Tumors from *BRCA1/2* non-carrier have significantly higher methylation at soloWCGW sites within partially methylated domains

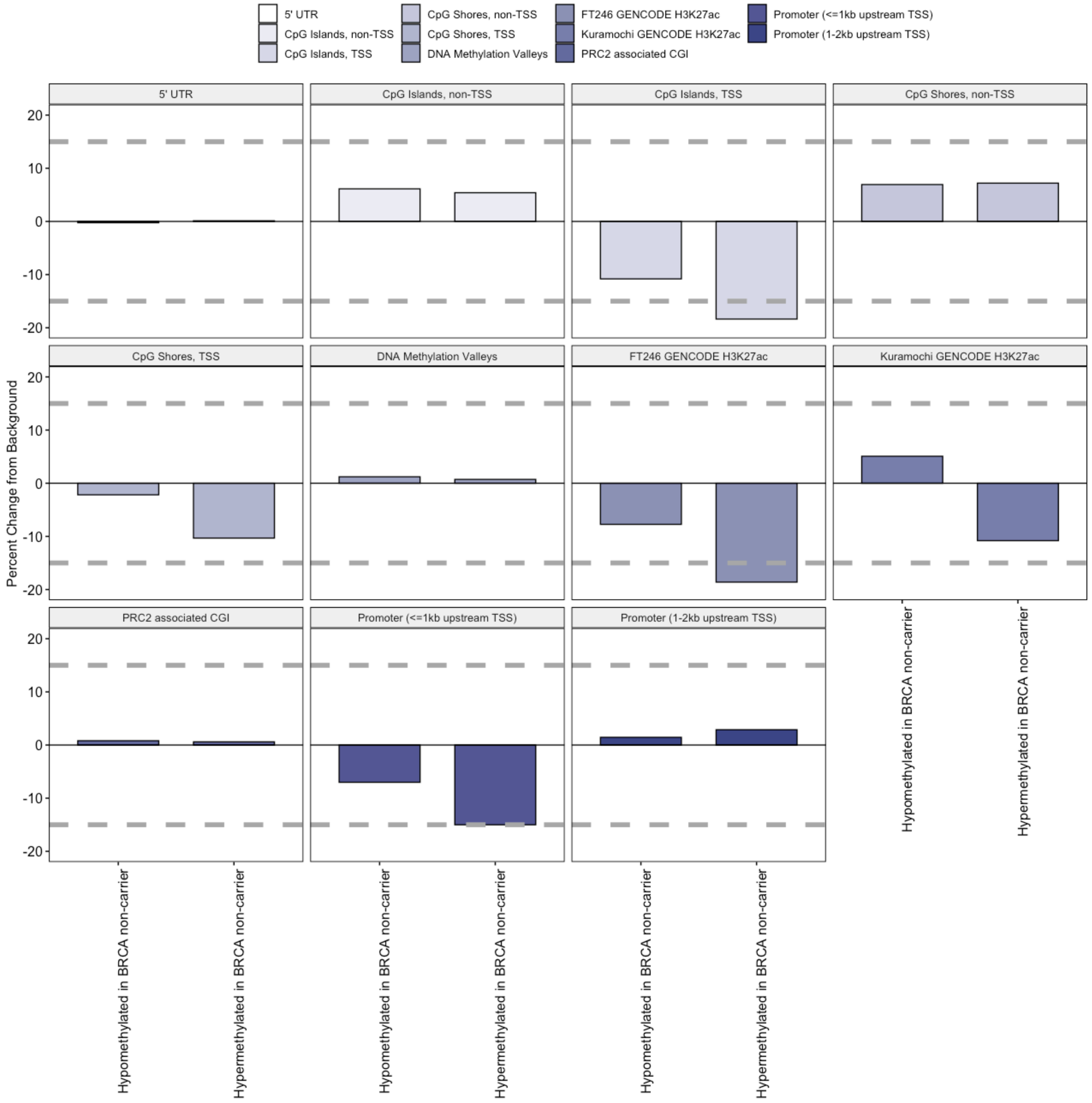

Supplementary Figure 9. Enrichment of differentially methylated regions (DMRs) in tumors from *BRCA1/2* carriers vs non-carriers: Enrichment of differentially methylated regions between tumors from *BRCA1/2* carriers and non-carriers were compared to a background set of genomic regions, matching DMR length and CpG content. DMRs were considered enriched at regions where the percent change from background was >15%, indicated by dashed grey line. Regions hypermethylated in *BRCA1/2* non-carriers were enriched in CpG islands associated with transcription start sites (CpG_TSS) and FT246 H3K27ac peaks. No enrichment was found for either hyper- or hypomethylated regions in 5' untranslated regions (UTR), non-TSS CpG islands, non-TSS CpG shores, TSS CpG shores, DNA methylation valleys, Kuramochi H3K27ac peaks, PRC2 binding regions, or promoters 1-2kb upstream of TSS.
